# Investigation of Strength and Fatigue Life of Rubber Asphalt Mixture

**DOI:** 10.3390/ma13153325

**Published:** 2020-07-26

**Authors:** Jiang Yuan, Songtao Lv, Xinghai Peng, Lingyun You, Milkos Borges Cabrera

**Affiliations:** 1 National Engineering Laboratory of Highway Maintenance Technology, Changsha University of Science and Technology, Changsha 410004, Hunan, China; yj@stu.csust.edu.cn (J.Y.); pengxinghsi@stu.csust.edu.cn (X.P.); combigra2013@gmail.com (M.B.C.); 2 Department of Civil and Environmental Engineering, Michigan Technological University, Houghton, MI 49931-1295, USA; liyou@mtu.edu

**Keywords:** rubber asphalt mixture, strength, fatigue life, genetic algorithm, back propagation neural network

## Abstract

Strength and fatigue life are essential parameters of pavement structure design. To accurately determine the pavement structure resistance of rubber asphalt mixture, the strength tests at various temperatures, loading rate, and fatigue tests at different stress levels were conducted in this research. Based on the proposed experiments, the change law of rubber asphalt mixture strength with different temperatures and loading rates was revealed. The phenomenological fatigue equation of rubber asphalt mixture was established. The genetic algorithm optimized backpropagation neural network (GA-BPNN) is highly reliable for optimizing production processes in civil engineering, and it has a remarkable application effect. A GA-BPNN strength and fatigue life prediction model was created in this study. The reliability of the prediction model was verified through experiments. The results showed that the rubber asphalt mixture strength decreases and increases with the increase of temperature and loading rate, respectively. The goodness of fit of the rubber asphalt mixture strength and fatigue life prediction model based on the GA-BPNN could reach 0.989 and 0.998, respectively. The indicators of the fatigue life prediction model are superior to the conventional phenomenological fatigue equation model. The GA-BPNN provides an effective method for predicting the rubber asphalt mixture strength and fatigue life, which significantly improves the accuracy of the resistance design of the rubber asphalt pavement structure.

## 1. Introduction

Rubber asphalt mixture has excellent fatigue resistance and water stability, which is an excellent choice for road engineering [1,2,3,4]. However, the asphalt pavement is constantly affected by environmental temperature changes, vehicle load, and other factors during its service life. The irregular and repeated changes of strain stress for a long time will cause the gradual weakening of the entire pavement structure’s strength. According to the current statistics, many roads do not reach the design life, and the pavement will produce cracks and other early diseases, which lead to fatigue fracture damage [5,6,7,8].

It is necessary to consider the structural design, material design, construction quality control, and other aspects to solve the early damage of asphalt pavement. Most of the design methods of asphalt pavement structures in the world use the mechanics-experience method, which uses mechanical methods to calculate the load response of the pavement structure. It uses homogeneous and isotropic linear elastic mechanics as the mechanical model for structural response calculation [9,10,11,12,13]. The strength, stiffness, and fatigue parameters are essential parameters in calculating the load response and establishing the mechanical model, which plays a vital role in the design of the pavement structure. Strengthening the anti-fatigue design of the asphalt pavement structure can significantly reduce the damage caused by unreasonable design, extend the service life of asphalt pavement, and improve the pavement’s service performance. Therefore, the study on the anti-fatigue design of the asphalt pavement structure will make the design of asphalt pavement more practical, more scientific, and reasonable.

Strength is an essential parameter for designing the pavement structure, reflecting the anti-destructive ability of asphalt pavement [14]. Some scholars have studied the impact of rubber particle size, content, and gradation type on the rubber asphalt mixture strength [15,16,17,18,19]. Rubber asphalt mixture has viscoelastic characteristics. The temperature and loading rate have a significant impact on its strength. The strength characteristics of rubber asphalt mixtures obtained by using different test methods have different results due to various stress modes. Furthermore, the phenomenon of the random value of strength parameters in the design of asphalt pavement structure is produced, which leads to an inaccurate calculation of the resistance of the pavement structure. However, the strength test results directly affect the results of the fatigue test of the rubber asphalt mixture.

The fatigue performance of asphalt mixture is the research hotspot of asphalt pavement. The factors that affect the fatigue performance of asphalt mixture mainly include the test method, material factor, loading frequency, test temperature, etc. In terms of test methods, indoor tests are primarily used in the world, including the direct tensile test, indirect tensile test, and four-point bending test. The fatigue test results obtained by different test methods and different specimen sizes are different. In terms of material factors, Li et al. studied the influence of asphalt types and gradation types on the fatigue performance of asphalt mixtures [20]. They found that Styreneic Block Copolymers (SBS) modified asphalt has the best fatigue performance. Jiang et al. conducted fatigue tests on porous asphalt mixtures composed of different materials [21]. They studied the effects of porosity, asphalt–aggregate ratio, and water immersion on the fatigue characteristics of the asphalt mixture. The results showed that the impact of water immersion on the fatigue characteristics of porous asphalt mixtures is closely related to the asphalt–aggregate ratio. Pell uses sine waves for loading, and the frequency range is 80–2500 r/min [22]. The results showed that the higher the frequency, the longer the fatigue life. The test temperature has a significant effect on the fatigue performance of the asphalt mixture. The studies have shown that when using stress control, the lower the temperature, the longer the fatigue life [23]. When strain control is used, the fatigue life is less dependent on the temperature at low temperatures. As the temperature increases, the fatigue life increases. In summary, it can be found that many factors will affect the fatigue performance of asphalt mixtures, and the fatigue test results are a vital factor that determines the accuracy of the fatigue life prediction equation of asphalt mixtures.

Fatigue life is an important basis for studying the anti-fatigue performance of asphalt pavement materials. Meanwhile, the fatigue life prediction is a long-lasting topic. There are three methods to forecast the asphalt mixture fatigue life: the dissipation energy method, fracture mechanics method, and phenomenology method [24,25,26,27,28].

There is a part of research on the prediction of asphalt mixture fatigue life according to the principle of dissipated energy [29,30,31,32,33]. According to Van Dijk et al.’s research, fatigue life mainly depends on the dissipation modulus and energy consumption during the stress–strain cycle [34]. The mechanical properties of the asphalt mixture depend on how long the load is applied and the temperature when the pressure is applied [35]. Its complex modulus is composed of a storage modulus and loss modulus. The main characteristic of the dissipated energy method is that there is a unique relationship between the cumulative dissipated energy and fatigue life [36,37]. Other factors (e.g., test method, loading mode) have a negligible effect. In fact, the dissipated energy of each cycle of damage is not constant, due to the accumulation of cracks in the mixture samples while conducting the asphalt mixture fatigue test, but it gradually increases with the increase of cycle times. Therefore, using a dissipated energy method to predict the fatigue life will cause higher errors in practical applications.

The fracture mechanics method predicts the fatigue life of asphalt mixtures, according to P.C. Paris’ crack growth formula [38]. Fracture mechanics divides the fatigue failure process into the crack initiation and crack propagation stage [39]. It is a continuous process from the formation of the fatigue crack to the propagation of the crack. The initial crack will be produced after the asphalt mixture has experienced a long fatigue process, which means that the initiation life of a fatigue crack is very long [40]. However, the life of this stage is ignored in fracture mechanics. Therefore, it is unreasonable to adopt the fracture mechanics method to predict the asphalt mixture fatigue life.

The phenomenological method is a common way for predicting the fatigue life of asphalt mixtures [41]. The fatigue equations are obtained by fitting the fatigue test curves under different strain or stress levels. The asphalt mixtures’ fatigue life under different strain or stress levels is estimated by using this equation [42]. However, there is some discreteness in the estimated equation. Thus, it is particularly important to predict the fatigue life of asphalt mixtures accurately.

Meanwhile, many scholars have done numerous research studies in data prediction. The backpropagation neural network (BPNN) is one of the commonly used methods for data prediction. The BPNN has the ability of robust nonlinear mapping and has been widely used in civil engineering. Abdelkader et al. used BPNN to predict cement concrete’s compressive strength [43]. Kheradmandi et al. proposed to use the BP method to calculate the interlayer modulus [44]. Besides, the fatigue life and optimum asphalt content also can be predicted with the BPNN model. Xiao et al. used regression analysis and neural network methods to forecast the recycled rubber asphalt mixture’ fatigue life [45]. Although the BP NN has achieved many outstanding results in the field of civil engineering, it takes a long time to approximate the predicted value, resulting in a slow convergence speed of the network. On the other hand, there are few studies on the application of BPNN in the rubber asphalt mixture’s strength and fatigue properties. However, the genetic algorithm (GA) has better global searchability, and it can obtain the optimal global solution with faster convergence speed [46]. Therefore, to improve the optimization ability of the BPNN and reduce the possibility that BPNN falls into a local optimization, the GA-BPNN model by using GA optimization on a BPNN is established. Considering the above reasons, GA-BPNN will be used to forecast the strength and fatigue life of rubber asphalt mixture in this research.

In summary, the accurate prediction of the rubber asphalt mixture strength and fatigue life is essential for ensuring the scientific and reasonable anti-fatigue design of rubber asphalt pavement structures. Based on this, in this study, the change rule of the strength of rubber asphalt mixtures with different temperatures and loading rates is revealed. The fatigue test is conducted at different stress levels. The conventional phenomenological fatigue equation of a rubber asphalt mixture is established. The prediction model of rubber asphalt mixture strength and fatigue life is created based on a genetic algorithm optimized backpropagation neural network (GA-BPNN). The goodness of fit of the fatigue life prediction model is compared with that one of the conventional phenomenological fatigue equation models. In addition to the training data, the strength and fatigue tests are carried out to verify the feasibility of the model.

## 2. Methods

### 2.1. GA-BPNN

**Figure 1:**
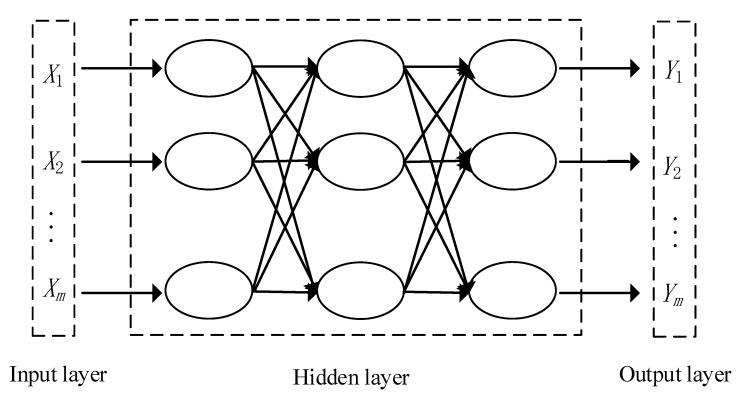
Backpropagation neural network (BPNN) structure.

BPNN is a kind of neural network algorithm based on error backpropagation [47]. Its training process is repeated alternately by forwarding propagation and backpropagation. The input, hidden, and output layers constitute the basic structure of the BPNN. Its basic structure is shown in Figure 1. *X*_1_, *X*_2_, …, *X_m_* are the input values. *Y*_1_, *Y*_2_, …, *Y_m_* are the output values.

GA is a computational model simulating the natural selection and genetic mechanism of Darwinian biological evolution [48]. The pattern theorem reveals the mechanism of GA., which is the main theorem of the genetic algorithm. The procedure is as follows.

Assuming that at a given time step *t*, a particular pattern *H* has *m* representative strings contained in population A(t), which is recorded as m=m(H,t). During the regeneration stage, the fitness of an individual $A_{i}$ is $f_{i}$, and each string replicates according to its fitness value. The regeneration probability of a string $A_{i}$ is:

(1) $$P_{i}=f_{i}/\sum_{i=1}^{n}f_{i}.$$

When non-overlapping *n* string populations are used instead of populations A(t), the following formula can be obtained:

(2) $$m\left(H,t+1\right)=m\left(H,t\right)nf\left(H\right)/\sum_{i=1}^{n}f_{i}$$

where f(H) is the average fitness of the string of pattern *H* in time *t*. The average fitness of the whole population can be recorded as:

(3) $$\bar{f}=\sum_{i}^{n}f_{i}/n.$$

Under the structural conditions of the basic GA, if the genetic operation only chooses to transfer to the next generation, the following formula holds:

(4) $$m\left(H,t+1\right)=m\left(H,t\right)f\left(H\right)/\bar{f}.$$

Equation (4) shows that a particular pattern grows according to the ratio between its average fitness value and the population’s average fitness value. Suppose that from t=0, the fitness value of a certain pattern is more than $c\bar{f}$ above the average fitness value of the population, and *c* is a constant. Then, the pattern selection growth equation becomes:

(5) $$m\left(H,t+1\right)=m\left(H,t\right)\frac{f\left(\bar{f}+c\bar{f}\right)}{\bar{f}}=\left(1+c\right)\cdot m\left(H,t\right)={\left(1+c\right)}^{t}\cdot m\left(H,0\right).$$

Under simple crossover, the probability of the general pattern *H* being destroyed is $P_{d}=\delta \left(H\right)/\left(t-1\right)$, and the survival probability is $P_{s}=1-\delta \left(H\right)/\left(t-1\right)$. The crossover itself occurs with a certain probability $P_{c}$, so the survival probability of pattern *H* is calculated as follows:

(6) $$P_{s}=1-P_{c}P_{d}=1-P_{c}\cdot \delta \left(H\right)/\left(t-1\right).$$

At the same time, considering the influence of selection and crossover operations on the pattern, an estimate of the sub generation pattern can be obtained:

(7) $$m\left(H,t+1\right)\geq m\left(H,t\right)\cdot \frac{f\left(H\right)}{\bar{f}}\cdot \left[1-P_{c}\frac{\delta \left(H\right)}{t-1}\right].$$

Suppose that the probability of a certain position of the string changing is $P_{m}$; then, the probability that the position is unchanged is $1-P_{m}$. The probability of pattern *H* being invariable is ${\left(1-P_{m}\right)}^{o\left(H\right)}$, where o(H) is the order of pattern *H*. The survival probability of pattern *H* under the action of mutation operator is:

(8) $$P_{s}={\left(1-P_{m}\right)}^{o\left(H\right)}\approx 1-o\left(H\right)P_{m}.$$

In summary, under the combined effect of genetic operator selection, crossover, and mutation, the number of samples of its offspring of pattern *H* are:

(9) $$m\left(H,t+1\right)\geq m\left(H,t\right)\cdot \frac{f\left(H\right)}{\bar{f}}\cdot \left[1-P_{c}\frac{\delta \left(H\right)}{t-1}\right]\cdot \left[1-o\left(H\right)P_{m}\right].$$

**Figure 2:**
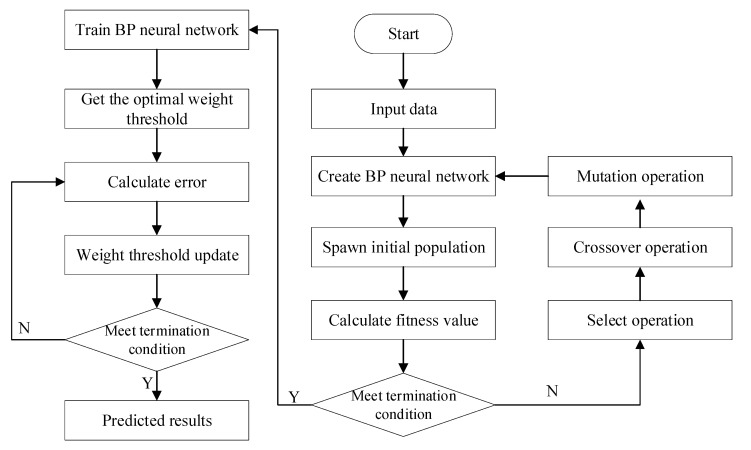
Flow chart of genetic algorithm optimized backpropagation neural network (GA-BPNN).

The GA is applied to the BPNN, and the weights and thresholds of the neural network are adjusted through the GA. Then, the weight and threshold are adjusted by using the BPNN to achieve better training results. The flow chart of GA-BPNN is shown in Figure 2.

In this paper, two nodes and one node are set in the input and output layer, respectively. By comparing the mean absolute error (*MAE*), it is finally determined that five nodes are set in the hidden layer. The transfer function of the output layer uses the Purlin function. The transfer function of the hidden layer uses the Tansig function. The Levenberg–Marquardt algorithm is used as the training function, which effectively overcomes the shortcomings of the slow convergence of the neural network. The learning rate is 0.5, the number of training steps is 1000, and the learning goal is 0.0001. The optimization parameter, maximum genetic generation, crossover probability, and mutation probability related to GA are equal to 30, 150, 0.6, and 0.05, respectively. All neural network simulations are performed on MATLAB R2016b.

### 2.2. Phenomenological Method

In the phenomenological method, the fatigue strength is the value of the repeated stress at which fatigue failure occurs in a material. The following is the derivation process of the fatigue equation based on the phenomenological method.

Considering the influence of stress amplitude, a common damage evolution model is [49]:

(10) $$\frac{dD}{dN}={\left(\frac{\sigma }{2M}\right)}^{\alpha }{\left(1-D\right)}^{-(\alpha +\gamma )}={\left(\frac{1}{2M}\right)}^{\alpha }{\left(\frac{\sigma }{1-D}\right)}^{\alpha }{\left(1-D\right)}^{-\gamma }.$$

Integrating Equation (10) obtains:

(11) $$D\left(N\right)=1-{\left(1-\frac{N}{N_{f}}\right)}^{\frac{1}{1+\alpha +\gamma }}$$

where the *N_f_* is the fatigue life, which can be expressed by Equation (12):

(12) $$N_{f}=\frac{1}{1+\alpha +\gamma }{\left(\frac{\sigma }{2M}\right)}^{\left(-\alpha \right)}=\frac{1}{\left(1+\alpha +\gamma \right){\left(2M\right)}^{-\alpha }}{\left(\frac{1}{\sigma }\right)}^{\alpha }.$$

Let $k=\frac{1}{\left(1+\alpha +\gamma \right){\left(2B\right)}^{-\alpha }},n=\alpha$, Equation (12) can be transformed into Equation (13).

(13) $$N_{f}=k{\left(\frac{1}{\sigma }\right)}^{n}$$

Equation (13) can be derived as follows:

(14) $$\mathrm{lg}N_{f}=\mathrm{lg}k-n\mathrm{lg}\sigma .$$

Equation (14) is a conventional phenomenological fatigue equation. Where σ is stress level; D is fatigue damage; N is times of loading actions; $N_{f}$ is fatigue life; and M,α,γ are material parameters to temperature.

## 3. Experimental

### 3.1. Materials

#### 3.1.1. Asphalt

In this paper, Donghai brand 70-grade road asphalt was selected as the neat asphalt. The rubber powder obtained from bias tires was sieved by using 40 mesh. The content of waste tire rubber powder is 21% of the neat asphalt mass. A small indoor mixer was used to prepare the rubber asphalt. The stirring temperature was set at 185 °C, and the stirring time was equal to 60 min. The main technical indexes of neat asphalt, waste tire rubber powder, and rubber asphalt are shown in Table 1, Table 2 and Table 3, respectively.

#### 3.1.2. Coarse and Fine Aggregate

Limestone was used for coarse and fine aggregate. The aggregate performance test results are shown in Table 4 and Table 5. Limestone mineral powder was used as filler.

#### 3.1.3. Grading Design

**Figure 3:**
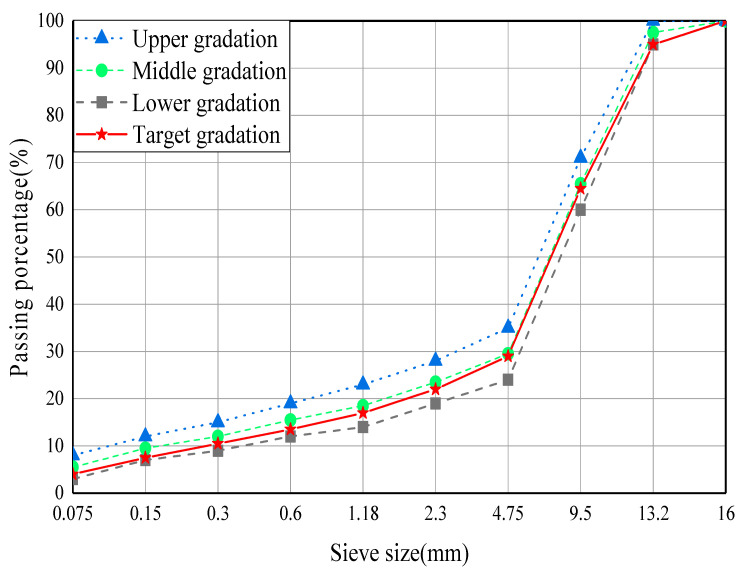
Gradation of the ARAC-13 rubber asphalt mixture.

According to the middle value of ARAC-13 type grading recommended in Shanghai 2012 technical code for rubber asphalt pavement, the grading curve in this paper is determined, as shown in Figure 3. The optimum asphalt content of rubber asphalt mixture is determined to be 7.2% by using the Marshall design method.

### 3.2. Experiment Scheme

To assess the tensile, compression, bending, and shear characteristics of asphalt mixtures, the commonly used methods for indoor strength tests include the direct stretching, uniaxial compression, four-point bending, and indirect stretching methods. There are many methods for fatigue properties testing. The most common test methods used across the world are the indirect tension method, trapezoidal cantilever bending method, and four-point bending method.

**Figure 4:**
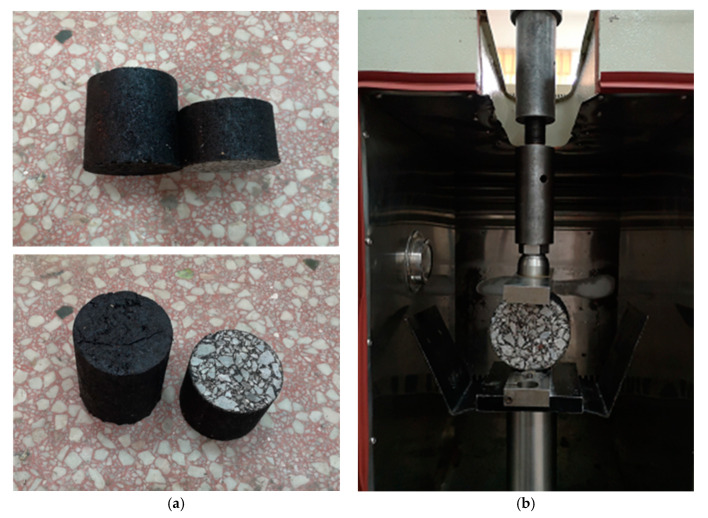
Indirect tensile test specimen and test drawing: (**a**) Rotary compacted cylindrical specimen; (**b**) Indirect tensile test chart.

Since the center point of the specimen in the indirect tensile test is in transverse tension and longitudinal compression, which is more consistent with the actual stress state of the asphalt pavement structure, the indirect tensile method was selected to study the strength and fatigue properties of rubber asphalt mixture in this research. The cylinder specimen with a diameter of 100 ± 2 mm and a height of 100 ± 2 mm was obtained by using rotary compaction. Then, the cylinder specimen with a diameter of 100 ± 2 mm and a height of 65 ± 2 mm was cut by utilizing a cutting machine. The specimen is shown in Figure 4a.

In this study, the strength and fatigue tests of rubber asphalt mixture are carried out with a MTS-Landmark operating system and the matching temperature control box. The MTS-Landmark operating system can provide different loading rates and stress levels, and the temperature control box can meet the requirements of the test temperature. Figure 4b shows the indirect tensile test.

Stress control was adopted in the strength and fatigue test of rubber asphalt mixture. The test temperatures for strength tests were equal to10 °C, 15 °C, and 20 °C. The loading rates were equal to 0.02 MPa/s, 0.05 MPa/s, 0.1 MPa/s, 0.2 MPa/s, 0.5 MPa/s, 1 MPa/s, and 2 MPa/s. The fatigue test temperature, the loading frequency, and the loading waveform are 20 °C, 10 Hz, and continuous positive vector waves, respectively.

## 4. Results and Discussion

### 4.1. Analysis of Strength Test Results

According to the experiment scheme, three parallel tests were conducted under the same conditions. The rubber asphalt mixture strength test results under different temperatures and loading rates are shown in Table 6.

**Figure 5:**
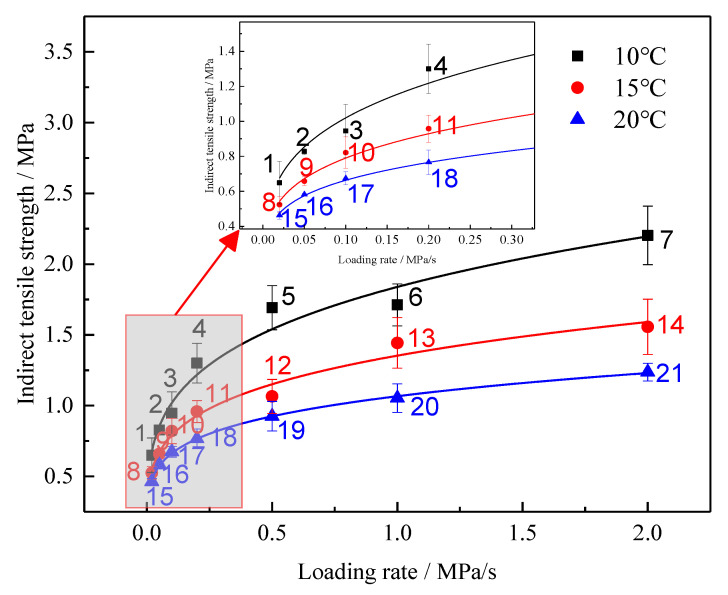
Law of changes related to indirect tensile strength with different loading rates and temperatures.

To analyze the influence of different temperatures and loading rates on the rubber asphalt mixture strength, the test results are presented in Figure 5 with a scatter diagram. It can be observed from Figure 5 that the strength value of rubber asphalt mixture increases with the increase of loading rate regardless of the temperature, and it decreases with the increase of temperature regardless of the loading rate. To better analyze the change rule of the rubber asphalt mixture strength, a nonlinear fitting has been carried out for the loading rate and strength. The fitting equation is as shown in Equation (15). The fitting results are shown in Table 7.

(15) $$S=\alpha \times v^{\beta }$$

where *S* is the strength value; *v* is the loading rate; α,β is the fitting parameter.

The results indicate that there is a good correlation between the indirect tensile strength and the loading rate at each temperature. It can be noticed from Table 7 that the change rule α is consistent with the change rule of rubber asphalt mixture strength, and both decrease with the increase of temperature. That is to say, α reflects the law of strength change. β reflects the sensitivity of the strength to the loading rate. The larger β is, the more sensitive strength is to the loading rate. It can be noticed from the analysis that while the temperature increases from 10 °C to 20 °C, β decreases gradually, and the sensitivity of the strength to the loading rate gradually decreases.

On the other hand, as the loading rate increases, the strength value increases; subsequently, its variability becomes greater and greater, and it is more susceptible to other factors. Yu et al. conducted the compressive resilience modulus test at 15 °C and 20 °C and the splitting strength test at 15 °C on three rubber asphalt mixtures [50]. The results showed that the splitting strength of different grade rubber asphalt mixtures has a special proportional relationship with the compressive resilience modulus. The difference in the rubber asphalt mixture between the two test temperatures is 0.85 times. Compared with neat asphalt mixtures, rubber asphalt mixtures have higher strength values. The test results of this research show that under the combined effect of test temperature and loading rate, the maximum strength value of rubber asphalt mixture, 2.203 MPa, is 4.75 times the minimum value, 0.463 MPa.

### 4.2. Establishment of the Strength Prediction Model

**Figure 6:**
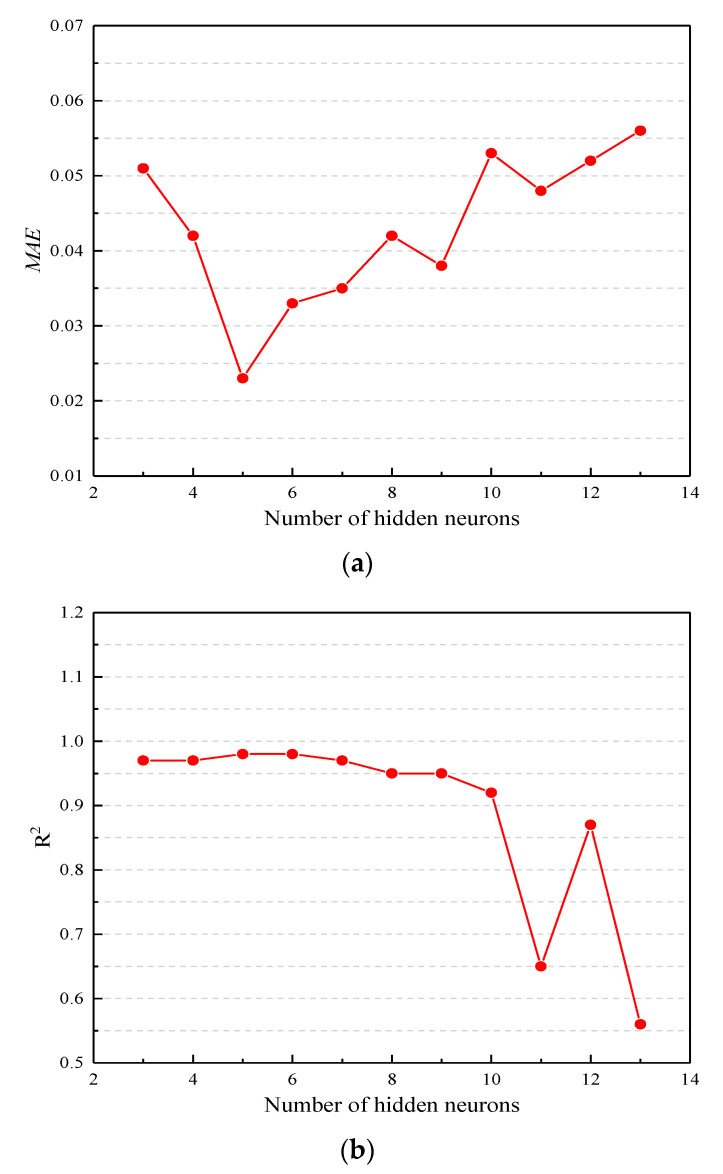

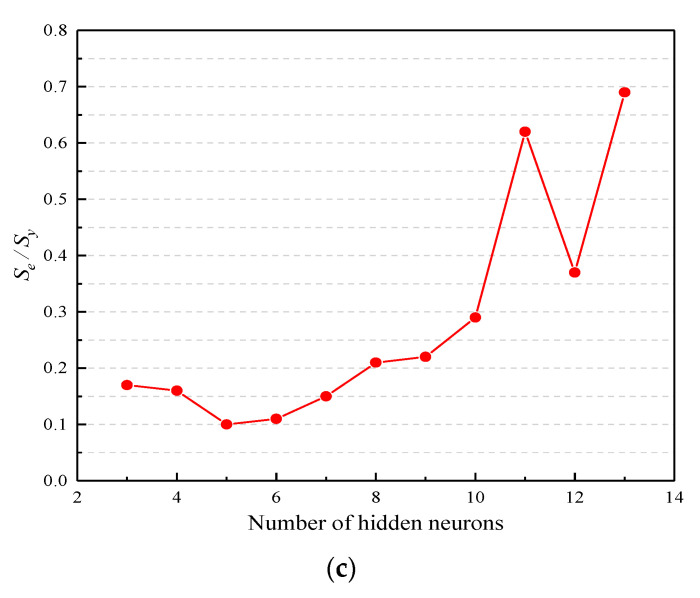
The results of the network structure with a different number of hidden layers: (**a**) The relationship between the mean absolute error (MAE) and the number of hidden neurons; (**b**) The relationship between R^2^ and the number of hidden neurons; (**c**) The relationship between Se/Sy and the number of hidden neurons.

The strength test data in Table 6 are processed, which obtain 21 group data required for training. Each set of training data contains 2 input values (temperature, loading rate) and 1 output value (average strength value). To increase the training speed, it is also necessary to normalize the data. The MATLAB R2016b program was used to build the GA-BPNN structure, as described above. Twenty-one sets of training data were used to train the network. There is no specific method for selecting the number of hidden layer nodes in the BPNN. If the number of hidden layer nodes is too small, it can lead to problems related to meeting the expected requirements, but too many hidden layer nodes may result in a lengthy training time and “overfitting”. In this study, to improve the accuracy of the model, different network structures were tried in the hidden layer. According to the general hidden layer determination method, the range of hidden layer nodes was selected from 3 to 13. Then, the number of hidden layer nodes was determined by comparing the mean absolute error (*MAE*), the goodness of fit (*R*^2^), and the standard error of predicted values divided by that of actual values (*S_e_/S_y_*). The calculation of *MAE*, *R*^2^, and *S_e_/S_y_* is as shown in Equations (16)–(18). Figure 6a–c reflect the results of the network structure corresponding to the hidden layer with a different number of nodes. The lower the *MAE*, the greater the *R*^2^, the lower the *S_e_/S_y_*, and the more accurate the prediction model. From Figure 6a–c, it can be found that when the number of hidden neurons is equal to 5, the value of *MAE* is the smallest; when the number of hidden neurons is equal to 5 and 6, the value of *R*^2^ is the largest. When the number of hidden neurons is equal to 5, the value of *S_e_/S_y_* is the smallest. It can be noticed from Figure 6 that while the number of hidden layer nodes is 5, the GA-BPNN structure achieves its best performance.

(16) $$MAE=\frac{1}{n}\sum_{i=1}^{n}\left(|Y_{pre}-Y_{exp}|\right)$$

(17) $$R^{2}=1-\frac{\sum_{i=1}^{n}{\left(Y_{pre(i)}-Y_{exp}{}_{\left(i\right)}\right)}^{2}}{\sum_{i=1}^{n}{\left(Y_{pre(i)}-\bar{Y_{exp}}\right)}^{2}}$$

(18) $$\frac{S_{e}}{S_{y}}=\sqrt{\frac{\sum_{i=1}^{n}{\left(Y_{exp(i)}-Y_{pre}{}_{\left(i\right)}\right)}^{2}\times n}{\sum_{i=1}^{n}{\left(Y_{exp(i)}-\bar{Y_{exp}}\right)}^{2}\times \left(n-m\right)}}$$

where $Y_{pre}$ is the predicted value; $Y_{exp}$ is the experiment value; and *n*, *m* are the number of tests and variables, respectively.

**Figure 7:**
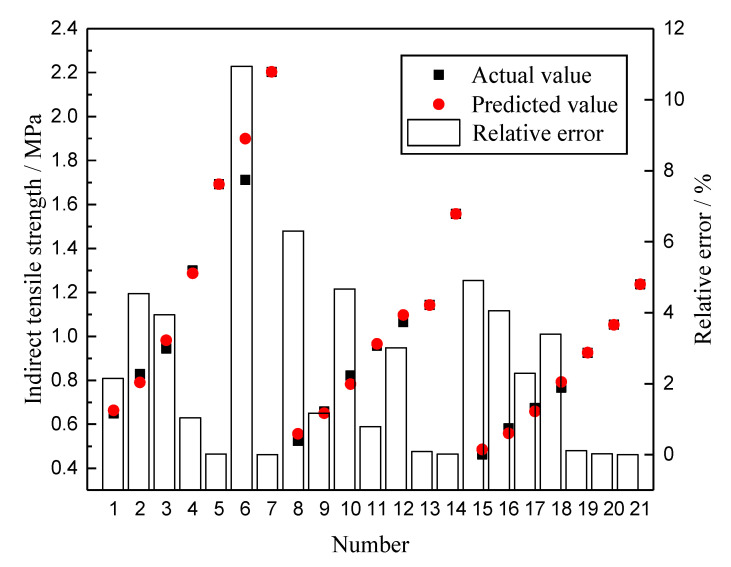
Prediction results of GA-BPNN.

By using the sim function, the trained network is simulated. The comparison between the predicted value of the GA-BPNN and the actual value of the test is shown in Figure 7. To facilitate the evaluation of the prediction results of the GA-BPNN model, Figure 7 shows the three variables of the actual value of the test, the predicted value, and the relative error in the same chart according to different sample numbers. The relative error is the absolute value of the difference between the actual value and the predicted value divided by the actual value.

**Figure 8:**
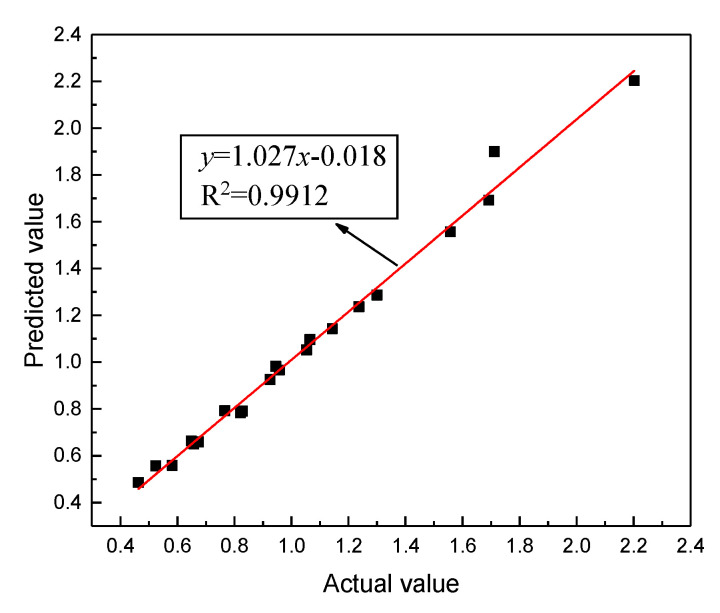
Fitting results of actual value and predicted value.

As can be seen from Figure 7, all predicted values almost completely overlap with the actual values of the experiment. The reliability of the prediction model is evaluated by calculating the mean square error (*MSE*), *R*^2^, and *S_e_/S_y_*. The calculation method of *MSE* is shown in Equation (19). It is calculated that the *MSE* is 0.002, the *R*^2^ is 0.989, and the *S_e_/S_y_* is 0.10. This shows that GA-BPNN can be used to forecast the strength of rubber asphalt mixture to achieve a good fitting result. Among the 21 sets of data, the relative error is within 10%, and the maximum relative error is 6.30%. These results further show that the GA-BPNN can guarantee the prediction effect for each individual data point. The predicted value and actual value are linearly fitted. The fitting result is shown in Figure 8. It can be inferred that GA-BPNN has a strong correlation between the input and output layers.

(19) $$MSE=\frac{1}{n}{\sum_{i=1}^{n}\left(Y_{pre}-Y_{exp}\right)}^{2}$$

where $Y_{pre}$ is the predicted value; $Y_{exp}$ is the experiment value; and *n* is the number of tests.

### 4.3. Analysis of Fatigue Test Results

Firstly, the rubber asphalt mixture strength test was carried out at the loading rate of 50 mm/min. The strength test results are shown in Table 8.

According to the strength test, it can be concluded that this parameter value is 0.729 MPa, and the failure load is 7.549 kN. The indirect tensile fatigue test of the rubber asphalt mixture was conducted under different stress levels. Four parallel tests are arranged under the same conditions. The rubber asphalt mixture fatigue test results are shown in Table 9.

**Figure 9:**
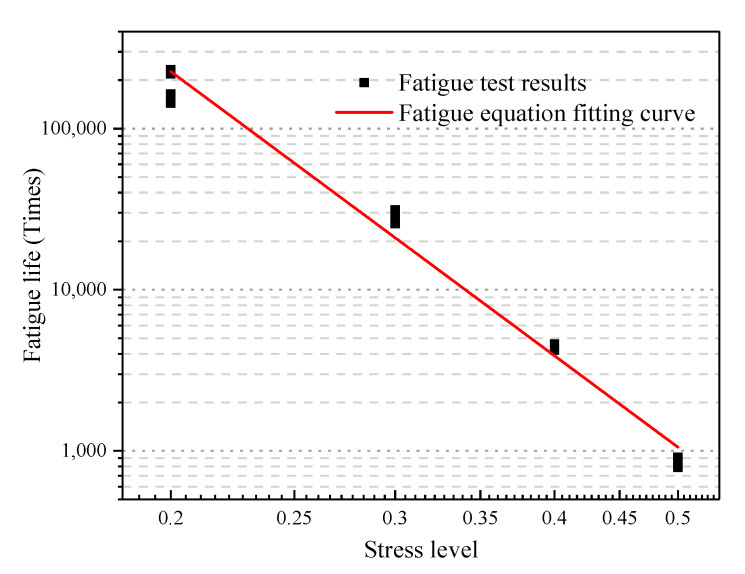
The law of fatigue life changing with the stress level.

The stress levels and fatigue life test results in Table 9 are presented in a double logarithmic coordinate system. According to Equation (14), the conventional phenomenological fatigue equation is obtained. The fitting result is shown in Figure 9. In Equation (13), the fatigue performance of a rubber asphalt mixture is reflected by two parameters, *k* and *n*. The value of *n* expresses the sensitivity of fatigue life to stress levels. The larger the value of *n*, the more sensitive the fatigue life is to the change of stress level. The value of *k* expresses the level of the line position of the fatigue curve. The larger the value of *k*, the better the fatigue durability. According to the fitting result of the fatigue test, k=18.16, and n=5.858. The phenomenological fatigue life equation is shown in Equation (20). The results show that the rubber asphalt mixture fatigue life gradually decreases with the increase in stress level.

(20) $$\mathrm{lg}N_{f}=1.259-5.858\mathrm{lg}\sigma$$

where $N_{f}$ is the rubber asphalt mixture fatigue life; and σ is stress level.

### 4.4. Establishment of the Fatigue Life Prediction Model

According to the GA-BPNN described above, the rubber asphalt mixture fatigue life was predicted. Each set contains two input values (stress level, stress ratio) and one output value (average fatigue life) for a total of 16 sets of training data. The number of hidden layer nodes is 5. To compare the prediction results of GA-BPNN and the conventional phenomenological fatigue equation, the fatigue life values are in logarithmic form. The prediction results of GA-BPNN are shown in Table 10. It can be noticed from Table 10 that the GA-BPNN model is more accurate in forecasting the rubber asphalt mixture fatigue life, and the relative error is smaller.

It is more persuasive to use a quantitative index to evaluate the prediction results. The predicted values of the two models are compared by using the *R*^2^, *MAE*, *MSE*, and *S_e_/S_y_*. The comparison results are shown in Table 11. It can be noticed that the *R*^2^ of the GA-BPNN model is better than that of the conventional phenomenological fatigue equation model. Besides, the *MAE* (2.371%), *MSE* (0.09%), and *S_e_/S_y_* (0.186) associated with GA-BPNN have superior values than those related to the conventional phenomenological fatigue equation. Therefore, all indexes show that the fitting effect of the GA-BPNN is superior to that of the conventional phenomenological fatigue equation.

Xie et al. predicted the fatigue performance of asphalt mixture based on BP neural network, and the goodness of fit of the model was 0.91 [51]. Yan et al. predicted the fatigue life of materials based on GA-BPNN, and the relative errors between the prediction results and the test data were less than 5% [52]. Compared with the existing research results, it can be found that the prediction accuracy of GA-BPNN for predicting the fatigue life of rubber asphalt mixture is better than the traditional prediction equation. It can be used as an effective method to obtain the fatigue life data of rubber asphalt mixture.

### 4.5. Validation of Strength and Fatigue Prediction Models

**Figure 10:**
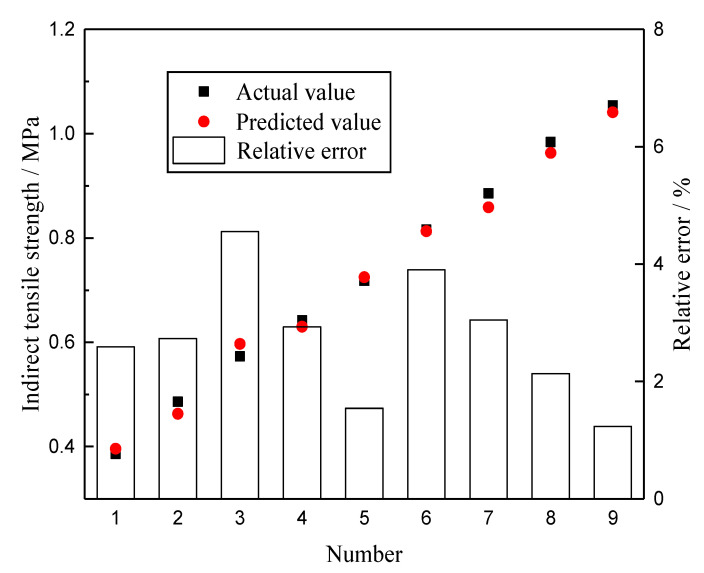
Validation results of the strength prediction model.

**Figure 11:**
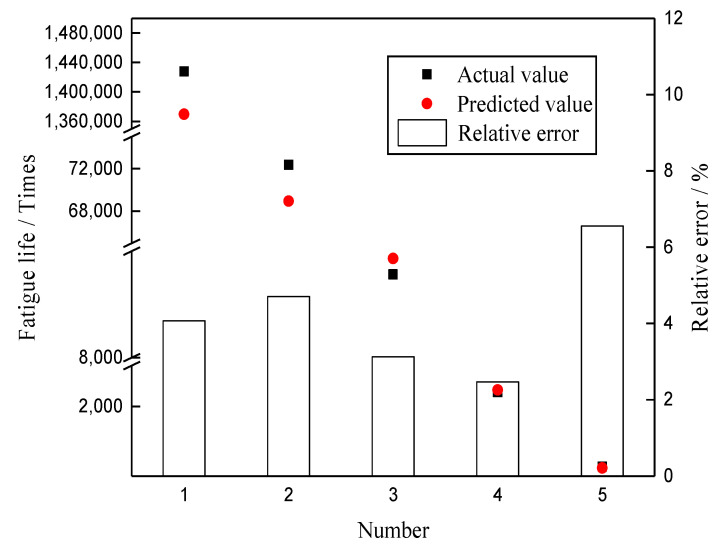
Validation results of fatigue life prediction model.

To verify the feasibility of GA-BPNN in the mechanical performance of rubber asphalt mixtures, the strength of this type of mixtures with different loading rates at 25 °C and its fatigue life under different stress levels were forecasted by using the established model. Indirect tensile strength and fatigue tests were carried out. The test results and predicted results are shown in Table 12 and Table 13, and Figure 10 and Figure 11. It can be found that the strength and fatigue life obtained from the tests are very close to the predicted value of the model, and the maximum relative errors are 4.553% and 6.554%, respectively. This result shows that it is feasible to forecast the strength and fatigue life of the rubber asphalt mixture by using GP-BPNN.

## 5. Conclusions

At present, the research on the asphalt mixtures strength and fatigue life prediction still mostly use strength or fatigue equations to regress the data. The asphalt mixtures fatigue damage is an extremely complicated process. It is difficult for conventional prediction models to achieve accurate prediction and prevention. In this paper, the prediction model of the strength and fatigue life of rubber asphalt mixture is established by using the GA-BPNN. The fatigue life prediction model and the conventional phenomenological fatigue equation model for forecasting the fatigue life of rubber asphalt mixture are compared. Experiments verify the reliability of the GA-BPNN prediction model. This paper provides some new inspiration and ideas for the research fields of the strength and fatigue life of rubber asphalt mixture. According to the experimental results in this research, the following main conclusions can be drawn.

(1) Based on the data of the indirect tensile strength and fatigue test, the GA-BPNN model is established. The goodness of fit of the model for predicting the strength and fatigue life of rubber asphalt mixtures can reach 0.989 and 0.998, respectively. The accuracy of the prediction model can meet the actual demand. The accurate prediction of rubber asphalt mixture strength and fatigue life can be realized.

(2) According to the four statistical indicators (*MAE*, *R*^2^, *MSE*, and *S_e_/S_y_*)_,_ the prediction effects of GA-BPNN and conventional phenomenological fatigue equation models were compared. The results showed that the indexes of the GA-BPNN model were superior to those of the conventional phenomenological fatigue equation model, which further improves the reliability of determining the fatigue resistance of rubber asphalt pavement structures.

(3) This study provides an effective method for predicting the strength and fatigue life of rubber asphalt mixtures. It offers reliable strength and fatigue design parameters for rubber asphalt pavement design, and it truly and effectively characterizes the structure resistance of the rubber asphalt pavement.

(4) This article only studies the influence of the temperature and loading rate on strength and stress levels in relation to fatigue life. The strength and fatigue life of asphalt mixtures are also affected by the material type, different test conditions, gradation, rubber powder content, and other factors. In the future, these other factors should be considered while estimating the fatigue life of the asphalt.

## Figures and Tables

**Table 1:** Indexes of neat asphalt.

Index	Unit	Test Results	Technical Requirement
Penetration (25 °C, 100 g, 5 s)	0.1 mm	64	60–80
Softening point	°C	48.5	≥46
Ductility (15 °C, 5 cm/min)	cm	>100	≥100
Density (15 °C)	g/cm^3^	1.029	-
Dynamic viscosity (60 °C)	Pa·s	199.3	≥180

**Table 2:** Technical indexes of waste tire rubber powder.

Index	Unit	Test Results	Technical Requirement
Water content	%	0.58	<1
Metal content	%	0.03	<0.05
Fiber content	%	0.03	<1
Ash content	%	6	≤8
Carbon black content	%	32	≥28
Rubber hydrocarbon content	%	55	≥42

**Table 3:** Technical indexes of rubber asphalt.

Index	Unit	Test Results	Technical Requirement
Penetration (25 °C, 100 g, 5 s)	0.1 mm	42.0	30–70
Softening point	°C	68.5	≥65
Ductility (15 °C, 5 cm/min)	cm	11.6	≥5
Dynamic viscosity (60 °C)	Pa·s	3.16	1.5–4
Elastic recovery	%	87.3	>60

**Table 4:** Test results of aggregate density.

Number	Particle Size Specification (mm)	Apparent Relative Density (g/cm^3^)	Bulk Volume Relative Density (g/cm^3^)	Water Absorption (%)
1	16–13.2	2.714	2.692	0.31
2	13.2–9.5	2.719	2.695	0.33
3	9.5–4.75	2.721	2.689	0.45
4	4.75–2.36	2.722	2.648	1.03
5	2.36–1.18	2.761	-	-
6	1.18–0.6	2.716		
7	0.6–0.3	2.726		
8	0.3–0.15	2.737		
9	0.15–0.075	2.725		

**Table 5:** Mechanical properties of limestone.

Aggregate Type	Crush Value (%)	Polishing Value (BPN)	Abrasion Value (%)
Limestone	15.8	57.4	19.9
Technical requirement	≤28	≥45	≤30

**Table 6:** The rubber asphalt mixture strength results.

Number	Temperature (°C)	Loading Rate (MPa/s)	Strength (MPa)			Mean Value of Strength (MPa)	Standard Deviations
			Sample 1	Sample 2	Sample 3		
1	10	0.02	0.520	0.762	0.665	0.649	9.94
2		0.05	0.846	0.811	0.827	0.828	1.43
3		0.1	0.852	1.120	0.863	0.945	12.38
4		0.2	1.429	1.150	1.321	1.300	0.11
5		0.5	1.794	1.512	1.770	1.692	0.13
6		1	1.651	1.881	1.604	1.712	0.12
7		2	2.394	1.984	2.231	2.203	0.17
8	15	0.02	0.516	0.484	0.572	0.524	0.04
9		0.05	0.680	0.662	0.632	0.658	0.02
10		0.1	0.758	0.782	0.923	0.821	0.07
11		0.2	1.044	0.893	0.937	0.958	0.06
12		0.5	0.970	1.025	1.200	1.065	0.10
13		1	1.512	1.577	1.240	1.443	0.15
14		2	1.594	1.346	1.731	1.557	0.16
15	20	0.02	0.447	0.454	0.488	0.463	0.02
16		0.05	0.584	0.573	0.589	0.582	0.01
17		0.1	0.682	0.633	0.707	0.674	0.03
18		0.2	0.845	0.713	0.740	0.766	0.06
19		0.5	1.016	0.948	0.811	0.925	0.09
20		1	1.128	0.939	1.092	1.053	0.08
21		2	1.308	1.191	1.212	1.237	0.05

**Table 7:** Fitting curve equation between indirect tensile strength and loading rate with different temperatures.

Temperature	α	β	*R* ^2^
10 °C	1.83887	0.25600	0.967
15 °C	1.35319	0.23358	0.974
20 °C	1.06645	0.20623	0.999

**Table 8:** The strength test results.

Number	Failure Load (kN)	Strength (MPa)	Average Strength (MPa)	Standard Deviation (MPa)	Coefficient of Variation
1	7715	0.745	0.729	0.015	0.021
2	3172	0.709			
3	7718	0.733			

**Table 9:** The rubber asphalt mixture fatigue test results.

Stress Level (MPa)	Stress Ratio	Fatigue Life (Times)				Average Fatigue Life (Times)	Standard Deviations
		Sample 1	Sample 2	Sample 3	Sample 4		
0.2	0.141	217991	164239	231877	143361	189367	36655
0.3	0.194	25702	27185	28941	31384	28303	2116
0.4	0.244	4215	4596	4410	4527	4437	144
0.5	0.292	849	789	912	810	840	47

**Table 10:** The fatigue life predicted results by using GA-BPNN.

Stress Level (MPa)	Stress Ratio	Fatigue Life Actual Value (Times)		Fatigue Life Predicted Value (Times)		Relative Error (%)	
		*N_f_*	Lg*N_f_*	*N_f_*	Lg*N_f_*	*N_f_*	Lg*N_f_*
0.2	0.274	189367	5.277	216489	5.335	14.322	0.101
0.3	0.041	28303	4.452	26444	4.422	6.568	0.673
0.4	0.549	4437	3.647	4384	3.642	1.195	0.137
0.5	0.686	840	2.924	829.50	2.919	1.250	1.385

**Table 11:** Comparison of model prediction effects.

Model	*R* ^2^	*MAE*/%	*MSE*/%	*S_e_/S_y_*
GA-BPNN	0.998	2.371	0.09	0.186
Conventional phenomenological fatigue equation	0.988	9.031	0.88	0.254

**Table 12:** Validation results of the strength prediction model.

Number	Temperature (°C)	Loading Rate (MPa/s)	Average Strength Test Value (MPa)	Predicted Strength Test Value (MPa)	Relative Error/%
1	25	0.01	0.386	0.396	2.591
2	25	0.03	0.486	0.463	2.731
3	25	0.08	0.573	0.597	4.553
4	25	0.15	0.642	0.630	2.928
5	25	0.3	0.718	0.725	1.541
6	25	0.6	0.816	0.813	3.901
7	25	0.8	0.886	0.859	3.047
8	25	1.5	0.984	0.963	2.134
9	25	2.5	1.054	1.041	1.233

**Table 13:** Verification results of the fatigue prediction model.

Stress Level	Average Fatigue Life Test Value (Times)	Predicted Fatigue Life Value (Times)	Relative Error/%
0.15	1427871	1369841	4.064
0.25	72358	68954	4.704
0.35	9566	9865	3.125
0.45	2348	2406	2.470
0.55	534	499	6.554
